# From deterministic to stochastic: limits of extracting bifurcation diagrams for noisy bistable oscillators with the control-based continuation method

**DOI:** 10.1007/s11071-024-10522-0

**Published:** 2024-11-11

**Authors:** Henrik T. Sykora, Sandor Beregi

**Affiliations:** 1 Department of Applied Mechanics, Budapest University of Technology and Economics, Budapest, Hungary; 2 Department of Infectious Disease Epidemiology, Imperial College London, London, UK

**Keywords:** Subcritical Hopf bifurcation, Noisy systems, Bifurcation diagram, Control-based continuation (CBC), Step-matrix multiplication path integral (SMM-PI) method

## Abstract

Noise limits the information that can be experimentally extracted from dynamical systems. In this study, we review the Control-based Continuation (CBC) approach, which is commonly used for experimental characterisation of nonlinear systems with coexisting stable and unstable steady states. The CBC technique, however, uses a deterministic framework, whereas in practice, almost all measurements are subject to some level of random perturbation, and the underlying dynamical system is inherently noisy. In order to discover what the CBC is capable of extracting from inherently noisy experiments, we study the Hopf normal form with quintic terms with additive noise. The bifurcation diagram of the deterministic core of this system is well-known, therefore the discrepancies introduced by noise can be easily assessed. First, we utilise the Step-Matrix Multiplication based Path Integral (SMM-PI) method to approximate the system’s steady state probability density function (PDF) for different intensity noise perturbations. We associate the local extrema of the resulting PDFs with limit cycles, and compare the resulting bifurcation diagram to those captured by CBC. We show that CBC estimates the bifurcation diagram of the noisy system well for noise intensities varying from small to moderate, and in practice, the amplitudes provided by CBC may be accepted as a ’best guess’ proxy for the vibration amplitudes characteristic to the near periodic solutions in a wide range of experiments.

## Introduction

Stochastic perturbations pose a major challenge in experimental bifurcation analysis, when the aim is to characterise the underlying nonlinear dynamics of real-life systems. Random perturbation to dynamical systems may induce uncertainty into measurement, and disguise, or in some cases, qualitatively alter the perceived dynamic behaviour compared to the equivalent deterministic case [1]. Nevertheless, for the description of dynamical systems, often deterministic differential equation models are preferred and identified. This creates a conundrum when system- or parameter identification is performed based on noisy data, since, it is unclear to what degree can one obtain information about the underlying deterministic dynamics [2], and it often requires a modelling hypothesis to fit deterministic models to data.

Stochastic nonlinear dynamical models are used in a wide range of fields. Indicative examples include problems from engineering, addressing vibrational problems such as aeroelastic flutter of aerofoils [3, 4], wheel-shimmy [5–7] or regenerative oscillations of machine-tools [8, 9], to climate modelling [10], biology [11] or computational medicine [12, 13] to capture various oscillatory phenomena. Each of these systems is usually subject to some level of random perturbation in practice. For example, aerofoils are generally affected by wind gusts and turbulence which is critical to account for in design, or, in machine tool vibrations, there is an expected fluctuation in the cutting force that affect the machined surface.

One of the most frequently observed behaviour in nonlinear dynamical systems is bifurcation: a change of system paramters results in qualitative change in the solution structure [14]. Our study focuses on one of the most common type of bifurcations, the Hopf bifurcation which describes the loss of stability of an equilibrium by oscillations while a set of limit cycles is born [15]. Since the Hopf bifurcation is a feature of autonomous systems, the related oscillations are often referred to as *self-excited* vibrations, or using a mechanical analogy, this may also be described as a *negative damping* in the system. The theory of Hopf bifurcations has been thoroughly investigated, and also significant results have been achieved in designing techniques to reduce complex or large-scale models (e.g., time-delayed, finite element, or fluid dynamical model) onto the centre manifold and obtaining the Hopf normal form [15, 16]. Another great amount of studies focus on *numerical continuation* tracing the branch of limit cycles (asymptotically stable or unstable periodic orbits) and the bifurcations related to these solution structures and several software packages, e.g., AUTO [17], CoCo [18], MatCont [19], DDEBiftool [20], have been developed which are capable of continuation of equilibria, limit cycles or quasi-periodic tori, in ordinary, delay or piecewise-smooth differential equations in one or multiple [21] bifurcation parameters.

In almost every application one can expect some level of unmodelled perturbation of the investigated system, perceived as random excitation. Therefore, it is unsurprising that the dynamics of stochastic nonlinear systems is of great interest in the scientific community. In [1] a dynamical system with additive noise is investigated, where the deterministic core of the model exhibits a pitch-fork bifurcation. This study describes fundamental dynamic behaviours which, as we will demonstrate, are also relevant in a nonlinear system with limit cycles. In [22], the local dynamics around a Hopf bifurcation in a system under random perturbation is studied. Our interest is the global system dynamics around subcritical Hopf bifurcations, i.e., when a bistable parameter domain is formed as the arising unstable branch of limit cycles undergoes a saddle-node bifurcation and folds back as a stable branch. This behaviour gives rise to a parameter region where a stable equilibrium and a stable limit cycle coexist in the phase space.

In bistable parameter-domains, depending on the noise level one may observe three types of behaviours: random vibration around one stable steady state solution, random oscillation with occasional jumps between domains of attractions and at very large noise levels, random vibration around more than one stable steady state solutions [1]. Here the stable steady state solutions refer to those of the underlying, unperturbed, deterministic system.

In the stochastic system, the concept of steady state stability is different. In particular, in bistable parameter domains no matter from which state ${\textbf {x}}(t_0)$ we start a trajectory at $t_0$, the first hitting time $t_{\textrm{eq}}:=\inf \{t\ge 0: {\textbf {x}}(t_0+t) \in \mathcal {B}_{\textrm{eq}}\}$ of the basin of attraction $\mathcal {B}_{\textrm{eq}}$ of the equilibrium and the first hitting time $t_{\textrm{lc}}:=\inf \{t\ge 0: {\textbf {x}}(t_0+t) \in \mathcal {B}_{\textrm{lc}}\}$ of the basin of attraction $\mathcal {B}_{\textrm{lc}}$ of the limit cycle will be both finite [23], i.e. $\mathbb {P}(t_{\textrm{eq}}<\infty ) = \mathbb {P}(t_{\textrm{lc}}<\infty ) = 1$. This means, that the the trajectories will eventually visit both steady states with probability one, even for small noise intensities. However, this result has little practical relevance, as the theory of large deviations [23] shows that the exit time from a basin of attraction of the stable steady states grows exponentially as the noise $\sigma \rightarrow 0$. Furthermore, due to the limitations of both the Monte-Carlo (MC) simulations and the SMM-PI method, for small noise intensities $\sigma _v$ we observe different steady state PDFs depending on the initial PDF of the initial states.

The simplest stochastic dynamical system producing this behaviour is described by the stochastic differential equation (SDE)

1 $$\begin{aligned} \textrm{d}{\textbf {x}} = {\textbf {f}}({\textbf {x}};\mu ) \textrm{d} t + \varvec{\sigma } \textrm{d}{W}, \end{aligned}$$

with $${\textbf {x}} = (u, v)^\top $$, $$\varvec{\sigma } = (0,\sigma _v)^\top $$ and

2 $$\begin{aligned} {\textbf {f}}({\textbf {x}};\mu )  &   = \begin{pmatrix} \mu &  \omega \\ -\omega &  \mu \end{pmatrix} \begin{pmatrix} u \\ v \end{pmatrix} + \left( u^2 + v^2\right) \begin{pmatrix} \alpha &  \beta \\ -\beta &  \alpha \end{pmatrix} \begin{pmatrix} u \\ v \end{pmatrix}\nonumber \\  &   \quad \ + \left( u^2 + v^2\right) ^2 \begin{pmatrix} \nu &  \gamma \\ -\gamma &  \nu \end{pmatrix} \begin{pmatrix} u \\ v \end{pmatrix}. \end{aligned}$$

Here *u* and *v* are the two state variables whereas $\mu , \omega , \alpha , \beta , \nu ,$ and $\gamma$ are scalar parameters, and $\sigma _v$ is the scalar noise intensity. In case $\sigma _v = 0$, system (1) describes the deterministic core, that is the Hopf normal form extended with quintic terms [15]. To investigate the effect of external random perturbations on such systems, we introduce an additive Gaussian white noise with the help of the standard Wiener process *W* and the noise intensity $\sigma _v$ to control the noise level. Note that restricting noise in the *v*-direction, even though this direction is generic in the system due to the rotational symmetry in the deterministic part, is a simplification of a realistic scenario where random perturbations may occur with varying direction. We still chose to make this compromise as unidirectional noise allows for a convenient quantification of the perturbation magnitude.

For deterministic nonlinear systems, such as the deterministic core in (1), the bifurcation diagrams are easily obtained by numerical continuation of the periodic solutions. Unfortunately, the established techniques of numerical continuation cannot be directly applied to systems with stochastic perturbation. We can overcome this limitation by using the popular measurement technique of control-based continuation (CBC) [24–26]. While the original intended use of CBC is in experiments, the technique can also handle complex numerical models where standard continuation algorithms are not feasible due to model scale or complexity [27] including examples where a nonlinear system is subjected to stochastic perturbation. However, as studies on stochastic nonlinear systems suggest (e.g. [4, 6, 28, 29]) that random perturbation is capable of modifying the solution structure of the systems. As such, it is unclear what CBC, which is an inherently deterministic method, actually reveals about the solution-structure of the stochastically perturbed system.

Our study aims to close this perceived information gap between the deterministic system, the real solution structure of the stochastic system and the one detected by CBC. To this end we obtain information about the global structure of the solutions through the probability density function (PDF) of the system’s steady state trajectories. A straightforward method to approximate the PDF is conducting excessive Monte-Carlo simulations. However, this technique has some limitations. Individual trajectories will be indicative of stable steady state solutions, whereas repelling solutions, which may still carry valuable information about the domains of attractions remain hidden. In principle, observing various extrema of the PDF can provide information of the steady state solutions in the system with maxima analogous to stable, while minima to unstable equilibria or limit cycles. An obvious limitation of this method is that maxima and minima can be inferred in an approximate sense only from discrete observations. Moreover, even though many models of stochastic perturbation, e.g. the Wiener process assuming a perturbation described by Gaussian white noise, have no limit on the magnitude of the perturbation, in practice, the probability of very large perturbations is extremely small. Therefore, there is no guarantee that a Monte-Carlo-simulation-based method, which has to be terminated in finite time, covers the full region of interest in the system’s phase-space. There are deterministic methods that resolve these issues. They rely on the solution of a partial differential equation (i.e. Fokker–Planck equation) or an integral equation (i.e. Chapman-Kolmogorov equation) corresponding to the response probability distribution function of the investigated system. In general, there is no explicit analytical solution for either equation, except for a small number of special cases, thus requiring a numerical approximation, such as [30, 31] or finite difference [32] methods. The main advantage of this approach over the MC method is that the results are deterministic, and as such, it is free from the perturbations that stochastic simulations introduce.

In this work, we choose to rely on the Step-Matrix Multiplication based Path Integral (SMM-PI) method [33], which approximates the steady state PDF of the system with high accuracy. This method relies on solving the Chapman–Kolmogorov (CK) equation [34] that describes the time evolution of the PDF of the system. The SMM-PI provides an approximate solution to the CK equation by transforming it into an iterative matrix multiplication. The resulting PDF solution is smooth and deterministic, therefore, finding its extrema is straightforward.

The rest of this paper is organised as follows. In Sect. 2 we summarise the deterministic Hopf normal form extended with quintic terms, discuss its bifurcation diagram, and give the draft of the CBC method. Next, Sect. 3 introduces the tools we use to analyse the effect of noise on nonlinear systems with bistable deterministic core. First, we adapt the CBC method for stochastic nonlinear systems, then introduce the SMM-PI method and how one may use the extrema of the steady state PDF as a surrogate of the bifurcation diagram. Then, in Sect. 4 we compare results obtained with SSM-PI to the bifurcation diagram obtained with CBC and assess the limitations of the CBC method in extracting information about the nonlinear system at different noise intensities. Finally, we conclude our findings in Sect. 5.

## Subcritical Hopf bifurcation in the deterministic system

### Bifurcation diagram

In this section we consider the behaviour of the deterministic core of the dynamical system (1) by setting $\sigma _v = 0$, reducing (1) to

3 $$\begin{aligned} \dot{{\textbf {x}}} = {\textbf {f}}({\textbf {x}};\mu ). \end{aligned}$$

To calculate equlibria and the amplitude of the limit cycles in this case it is common practice to switch to polar coordinates

4 $$\begin{aligned} u = r \cos \phi , \quad v = r \sin \phi , \end{aligned}$$

where *r* denotes the radius whereas $\phi$ stands for the phase angle. This provides an ODE for the radius

5 $$\begin{aligned} \dot{r} = \mu r + \alpha r^3 + \nu r^5, \end{aligned}$$

whereas one obtains $\dot{\phi } \approx - \omega$ for the phase angle. The system (5) has an equlibria in $r^{\textrm{eq}} = 0$ while the amplitude of the deterministic limit cycles in (1) with the help of the positive equilibria of Eq. (5), namely,

6 $$\begin{aligned} r^{\textrm{lc}}_{1,2} = \sqrt{\frac{-\alpha \pm \sqrt{\alpha ^2 - 4 \mu \nu }}{2 \nu }}. \end{aligned}$$

From (6) follows that the periodic orbits have a circular shape in the (*u*, *v*) phase-plane for the deterministic system. Thus, the system has rotational symmetry with respect to the origin $(u,v)=(0,0)$ and the equilibria $r^{\textrm{eq}}$ and $r^{\textrm{lc}}$ define the exact vibration amplitudes in *u* and *v*.

In order to determine the stability of each solution $r^{\textrm{eq}}$ and $r^{\textrm{lc}}$ we can consider the system (5) as a gradient system

7 $$\begin{aligned} \dot{r} = -\frac{\textrm{d}}{\textrm{d}{r}}U_{\mu }(r), \text{ with } U_{\mu }(r) = -\frac{\mu }{2} r^2 - \frac{\alpha }{4} r^4 - \frac{\nu }{6} r^6, \end{aligned}$$

where the stable equlibria are the minima of the potential $U_{\mu }(r)$, while the unstable equlibria are the maxima.

**Fig. 1:**
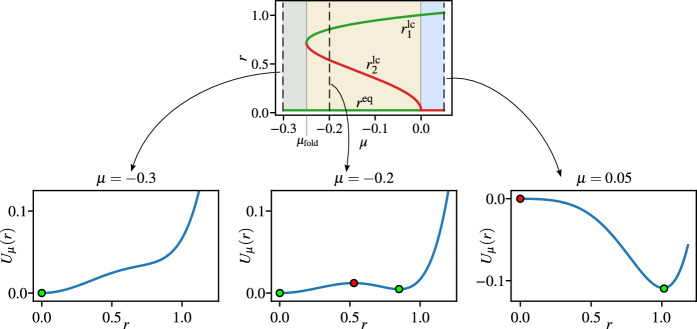
Diagram of the subrictical Hopf bifurcation for the deterministic system (3) with parameters $\omega =\alpha = \beta = \gamma = 1$ and $\nu = - 1$, and example potential functions $U_\mu$ for different $\mu$ values. The green and red dots represent the stable and unstable steady states, respectively

To ensure a subcritical Hopf bifurcation and bistability ($\alpha ^2 - 4 \mu \nu > 0$) we set $\alpha > 0$ and $\nu <0$. In this case, we have three distinct parameter regions: in case of $\mu \in (-\infty ,{\mu _{\textrm{fold}}}]$ we have a single stable equilibrium at $r^{\textrm{eq}} = 0$, if $\mu \in [0,\infty )$ we have a stable limit cycle at $r^{\textrm{lc}}_{1}$, while in case of $\mu \in [{\mu _{\textrm{fold}}},0]$ we have a stable equilibrium at $r^{\textrm{eq}} = 0$, an unstable limit cycle at $r^{\textrm{lc}}_{2}$, and a stable limit cycle at $r^{\textrm{lc}}_{1}$. We refer to the parameter $\mu _{\textrm{fold}} = \frac{\alpha ^2}{4 \nu }$ as the fold point as the solution-branch folds back above the unstable part continues with a higher amplitude stable branch. As we specifically focus on the effect of the parameter $\mu$, we restrict the parameters $\omega =\alpha = \beta = \gamma = 1$ and $\nu = - 1$. Figure 1 shows the three parameter regions along with example potentials $U_{\mu }(r)$ for each parameter regions.

### Control-based continuation

Control-based continuation is a technique to find limit cycles in physical experiments or in numerical models where it is not possible or practical to use boundary-value-problem-based solvers [24–26]. This method is especially powerful in finding unstable limit cycles in such systems. We trace these unstable steady state solutions in the system by applying a *stabilising* and *non-invasive* control law ${\textbf {F}}(t)$, leading to the system of the form

8 $$\begin{aligned} \dot{{\textbf {x}}} = f({\textbf {x}};\mu ) + {\textbf {F}}(t), \end{aligned}$$

To construct the appropriate control ${\textbf {F}}(t)$, first, we assume, that there exist a periodic solution ${\textbf {x}}_{\textrm{p}}(t)$ for the open-loop system. In system (3) we have such steady state solution, e.g. $u_{\textrm{p}}(t) = r \cos \phi (t)$. Applying a stabilising control law with $y(t) = A \cos {\phi (t)}$ as a periodic control target means that this control results in the controlled coordinate converging to a periodic solution $u(t) \rightarrow u^{*}(t) = A^*\cos {\phi (t)}$ near the control target *y*(*t*). Here, we use the term *non-invasive* to emphasize that if the steady state periodic solution $u^{*}(t)$ of the controlled system is equal to the target *y*(*t*), then it is also coincident with the limit cycle in the open-loop system, i.e. $u^{*}(t) = y(t) = u_\textrm{p}(t)$, and therefore, the steady state control force is zero. That is, the stabilising and non-invasive control does not introduce a new steady state, it only stabilises an existing unstable one. We can ensure this by selecting the appropriate control-target, that in the ideal case results in zero control input in steady state. In case of the Hopf normal form, with an appropriately chosen control gain *k*, considering a simple proportional error term

9 $$\begin{aligned} {\textbf {F}}(t) = \begin{pmatrix}k \left( A^*\cos {\phi (t)} - u(t)\right) \\ 0 \end{pmatrix} \end{aligned}$$

provides such a stabilising control-law. To find the appropriate control target $A^*$ that corresponds to the amplitude $r$ of the limit cycle we use iteration. It is generally assumed that $A^{*}$ is near *A*. This is ensured by starting the continuation from a stable branch in the bifurcation diagram, where solutions converge to *u*(*t*) without control. As such, the starting guess for the amplitude *A* is taken from a stable periodic solution. Thereafter the closeness of $A^{*}$ and *A* is ensured by providing further guesses based on previously identified solutions with a continuation algorithm. The full details of the process are given in [27]. In order to make an iteration possible, we consider the truncated Fourier series of the steady state solution of the controlled experiment, the control target and the limit cycle of the open-loop system. Thus, the problem of finding an appropriate time-varying control target is simplified into an algebraic root-finding problem where the aim is to obtain the appropriate Fourier coefficients of the control target. Further details of the CBC algorithm are provided in Appendix A.

## Hopf normal form with process noise

### Tracing limit cycles in presence of process-noise

To account for the effect of random perturbations one may observe in experiments, Gaussian white noise is added to the Hopf normal form with quintic terms as defined in Eqs. (1) and (2). While CBC in principle assumes that a deterministic dynamical system is surveyed, it may be applied to systems subject to random perturbation [26, 35]. In fact, this is the case in practically any physical experiment. Nevertheless, noise often has little effect on the dynamics of the observed system and, as such, is frequently ignored and the experiment is treated within a deterministic framework.

This study considers the case of *mild* but *non-negligible* noise, i.e, when the effect of process noise on the perceived dynamics is significant but still not too strong to entirely disguise the features of the deterministic core of the system. Thus, in place of the limit cycles, near-periodic solutions can be identified. The most distinctive feature of these solutions is that a varying vibration amplitude and period is perceived instead of the constant values defining the limit cycles in deterministic systems. Nevertheless, to adopt the CBC technique for the stochastic case, we will assign a single vibration amplitude, that is representative of the observed oscillations in the statistical sense, and still assume that the natural angular frequency is described by $\omega$, thus constructing a quasi-deterministic root-finding problem.

In the numerical SDE model, the characteristic vibration amplitude is obtained by performing Monte Carlo simulations on an ensemble of size *m*. We take the simulated solution segments $u_1^j(t),$ $t \in [t_0, t_M]$ corresponding to the $j^\textrm{th}$ realisation, where $t_0$ is chosen such that the transients are not included. This can be ensured by discarding the solutions and carrying on with the simulation while the transients decay. Then we define the characteristic vibration amplitude $\overline{A}_1$ as the minimiser of the mean square error

10 $$\begin{aligned} \overline{A}_1 := \underset{{A}_1\in \mathbb {R}^+}{\textrm{argmin}} \; \frac{1}{m} \sum _{j=1}^m \sum _{i=0}^M \left( u_{1i}^j - A_1 \cos \phi (t_i)\right) ^2, \end{aligned}$$

where $A_1 \cos \phi (t_i)$ is the base harmonic of the limit cycle. Then, the established characteristic amplitude is used in the error function $\overline{e}$

11 $$\begin{aligned} \overline{e} = A_\textrm{1t} - \overline{A}_1, \end{aligned}$$

while the control target is still considered as a periodic orbit as in the deterministic case.

Note that if the true probability density function (PDF) of the trajectories were known, this would be indeed a deterministic root-finding problem. This is due to the fact, that the stochastic process is assumed to be *ergodic*. Therefore, there is a steady state PDF with statistical moments constant in time. However, by Monte Carlo simulations we only consider a finite sample from the solution PDF. As such, the resulting characteristic amplitude will still show some variation, albeit to a significantly lesser degree as one would observe by studying the time-histories themselves. Therefore, in the continuation algorithm, we use a surrogate model of the noisy error function to avoid the direct calculation of derivatives in the true zero problem, which may show significant fluctuations making iterative root-finding impossible.

The continuation algorithm to trace the branch of near periodic solutions in the SDE with respect to system parameters is constructed in a similar fashion to the deterministic case, with the modification of using the statistical characteristic amplitude instead if the deterministic vibration amplitude. The details of the continuation algorithm are provided in the A.

### steady state probability density function of the stochastic Hopf normal form

To compute the steady state response PDF of the open-loop system (1) we use the Step Matrix Multipliation-based path integral (SMM-PI) method [33]. The method is based on the Chapman-Kolmogorov equation

12 $$\begin{aligned} p({\textbf {x}},t_{n+1}) = \int \limits _{\mathbb {R}^2} p({\textbf {x}},t_{n+1}|{\textbf {y}},t_n) p({\textbf {y}},t_n) \textrm{d}{\textbf {y}} \end{aligned}$$

that we solve iteratively until we reach a steady state PDF $p_{\textrm{st}}({\textbf {x}}) = \lim _{t \rightarrow \infty } p({\textbf {x}},t)$. Here, $p({\textbf {x}},t_{n+1}|{\textbf {y}},t_n)$ is the joint PDF of transitioning to state ${\textbf {x}}$ at time $t_{n+1}$ from state ${\textbf {y}}$ at time $t_n$.

The SMM-PI method estimates the time evolution of the PDF $p({\textbf {x}},t_n)$ by transforming (12) into the matrix–vector multiplication

13 $$\begin{aligned} \textrm{vec} \left( {\textbf {q}}_{n+1} \right) = {\textbf {S}}\, \textrm{vec} \left( {\textbf {q}}_{n}\right) , \end{aligned}$$

where ${\textbf {q}}_n\in \mathbb {R}^{N_u\times N_v}$ is a matrix containing the probability density function $q_{i,j,n}:= p([u_{i},v_{j}]^\top ,t_n)$ in the discrete points $\{u_{i},v_{j} | i = 1,\ldots ,N_x, j= 1, \ldots , N_v\}$ over a finite region $[u_1,u_{N_u}]\times [v_1,v_{N_v}]$ and ${\textbf {S}} \in \mathbb {R}^{{N_u N_v\times N_u N_v}}$ is the step matrix corresponding to the time step between $t_n$ and $t_{n+1}$ for $n = 0,1,2,\ldots$. This transformation has three key elements: (i) the approximation of the transitional probability density function (TPDF) $p({\textbf {x}},t_{n+1}|{\textbf {y}},t_n)$, (ii) the spatial discretization of the PDF $p({\textbf {x}},t_n)$, and (iii) the quadrature computation of the integral, all detailed in [33].

As the open-loop system (1) is time-invariant and we use a constant time step $\Delta t$ for each $n$, we have to compute the step matrix ${\textbf {S}}$ once. Then we advance the PDF $p({\textbf {x}},t)$ a single time-step via the matrix–vector multiplication in (13) which is one of the fastest methods to approximate (12) accurately [33].

Furthermore, if we have a sufficiently large noise intensity $\sigma _v$ (e.g. in case of (1) $\sigma _v>0.1$) then (13) has a single steady state ${\textbf {q}}:= \lim _{n\rightarrow \infty } {\textbf {q}}_n$. In this case the eigenvector ${\textbf {s}}_{(1)}$ of ${\textbf {S}}$ corresponding to the eigenvalue with the largest magnitude $\lambda _{(1)}\approx 1$ is the only surviving eigenvector following the successive multiplications in (13), and we find that ${\textbf {q}} = {\textbf {s}}_{(1)}$ represents the steady state PDF $p(x,v)$, given that the time step $\Delta t$ and resolutions $N_x$ and $N_v$ are sufficient for the approximation of (12). Therefore, throughout this paper we use this eigenvector to guarantee that we obtain the steady state solution ${\textbf {q}}$ of (13) corresponding to the steady state PDF $p(u,v):= \lim _{t\rightarrow \infty } p([u,v]^\top ,t)$.

## Results

### Steady state PDFs of the open-loop system

We first consider the stochastic open-loop system described by Eq. (1) and investigate its steady state behaviour. As discussed in Sect. 2.1, in the case when a bistable parameter domain appears ($\alpha >0$, $\nu <0$) the bifurcation model of the deterministic core of the system, defined by the Hopf normal form with quintic terms (see Eq. (3)), has three qualitatively different regions with respect to the bifurcation parameter $\mu$. For $\mu < \mu _\textrm{Fold}$, the deterministic system has a single stable equilibrium, for $\mu \in (\mu _\textrm{Fold}, 0)$, we have bistability, namely a stable equilibrium and a stable limit cycle separated by an unstable limit cycle, whereas for $\mu >0$ the system has an unstable equilibrium and a stable limit cycle. In the numerical examples, we consider the system parameters $\omega =\alpha = \beta = \gamma = 1$ and $\nu = - 1$. In this case the Fold point in the bifurcation diagram is at $\mu _\textrm{Fold} = -0.25$.

**Fig. 2:**
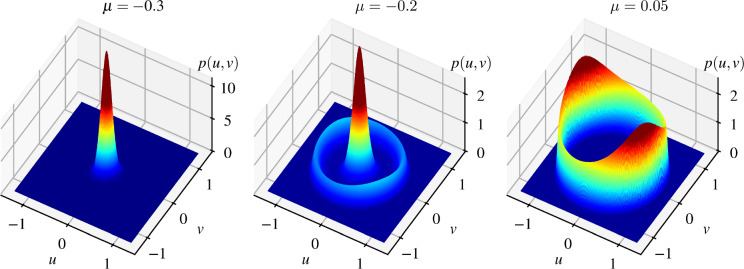
Examples of $p(u,v)$ for $\omega =\alpha = \beta = \gamma = 1$, $\nu = - 1$, $\sigma _v = 0.1$ and different values of the parameter $\mu$

**Fig. 3:**
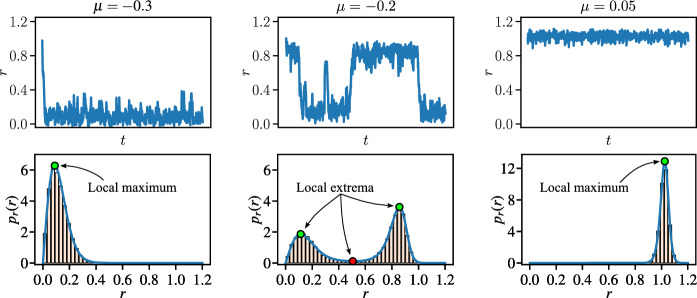
Example trajectories for $r = \sqrt{u^2+v^2}$ (top row) along with the corresponding radial PDFs $p_r$ with MC comparison denoted with the orange histogram (bottom row) at $\sigma _v=0.1$. The green and red dots represent the local maxima and minima of the PDF $p_r$, respectively

It is reasonable to expect that the three qualitatively different parameter regions will be preserved with a random perturbation with moderate intensity. This is indeed the case as demonstrated in Fig. 2 showing steady state PDFs of the open-loop system with a noise intensity $\sigma _v = 0.1$. In the case of $\mu = -0.3$ (left panel), where the deterministic core has a single stable equilibrium at $(u,v) = (0,0)$, we have a PDF with a single peak. For $\mu = 0.05$ (right panel), the additive noise causes the trajectories to oscillate near the stable limit cycle, and we observe a circular ridge in the PDF along this limit cycle (the equilibrium $(u,v) = (0,0)$ in the deterministic core is unstable). These results also coincide with the ones presented in [22, 36] considering the classical Hopf normal form up to 3rd order terms. However, for $\mu$ values where both the stable equilibrium and the stable limit cycle exist in the deterministic core, the solution either converges to the equilibrium or to the limit cycle. In the stochastic system we observe a slightly different behaviour [1, 23, 37, 38], namely the steady state PDF have both a peak and a ridge near the stable equilibrium and the stable limit cycle, respectively. This means that trajectories occasionally switch between the basins of attractions of the two steady states instead of staying near the equilibrium or the limit cycle.

This is best illustrated by the time evolution of the radius $r = \sqrt{u^2+v^2}$ in Fig. 3 corresponding to the three behaviours in Fig. 2. Here we also consider the one-dimensional PDFs $p_r(r)$ with respect to the polar-coordinate *r*:

14 $$\begin{aligned} p_r(r) = \int \limits _{-\pi }^{\pi } p(r \cos {\phi },r \sin {\phi })\, r\, \textrm{d}\phi . \end{aligned}$$

In Fig. 3, we show the radial PDFs $p_r$ obtained from the SMM-PI based PDF $p(u,v)$, corresponding to the trajectories. For validation, we compare the radial PDFs $p_r$ to the histograms obtained from the steady state solution segments ($t \in [99,100]$ sampled at 100 equidistant instants) of an ensemble of 1000 trajectories used.

Next, we use the local maxima of the radial PDFs $p_r$ as the characteristic amplitude of the corresponding solutions to compare them to the deterministic bifurcation diagrams. Notice that this definition of the characteristic amplitude may lead to different results as in the deterministic case. This is most prominent in the case of the single stable equilibrium ($\mu = -0.3$, bottom-left panel), where the stable equilibrium in the deterministic system would suggest zero amplitude, while, with noise, the peak is clearly at $r>0$. The discrepancy originates in how geometric probability is defined: if one considers the (*u*, *v*)-plane in polar coordinates, then increasing the radius *r* results in circles of increasing lengths from where probability is collected. As the origin is point-like, it has a radial PDF $p_r(0) = 0$. For an analogy, consider the popular game of throwing darts at a circular target. In this game, if one aims at the centre and considers random error in throws, then hitting the exact centre of the target (or any other specific point) also has zero geometric probability. Nevertheless, this amplitude definition is consistent with the amplitude commonly defined in bifurcation analysis and further, it results in a continuous transition between the steady state near the equilibrium of the deterministic system and the unstable limit cycle. With this definition, we can also view the deterministic case as the limiting behaviour of the stochastic system, where $\sigma _v \rightarrow 0$.

An additional benefit of using the PDFs is that one can also identify the local minima, which then can be associated with the unstable steady state solutions, which are normally ‘invisible’ in simulation or in an experiment without control. As outlined in the Introduction 1, the interpretation of unstable limit cycles in a stochastic system is not trivial as this structure fails to satisfy the criteria to be considered as a steady state (i.e. a trajectory started within stays in the said domain) due to the random perturbation. Instead, we look at another property of unstable limit cycles which can be transferred to stochastic systems, i.e. in the deterministic case, the unstable limit cycle corresponds to the separatrix between the domains of attractions of the stable equilibrium and the stable limit cycle. However, as explained in the discussion of the PDFs obtained for the bistable case (centre column of panels in Figs. 2 and 3), the basins of attractions of the two stable solutions are not isolated any more due to the noise perturbation introduced to the system. Nevertheless, one may still consider the path in the (*u*, *v*) phase-plane where the system is locally least likely to be, i.e. corresponding to a local minimum of the PDF. Thus, even though they do not define perfect barriers, they are analogous to the unstable limit cycles in the deterministic system.

**Fig. 4:**
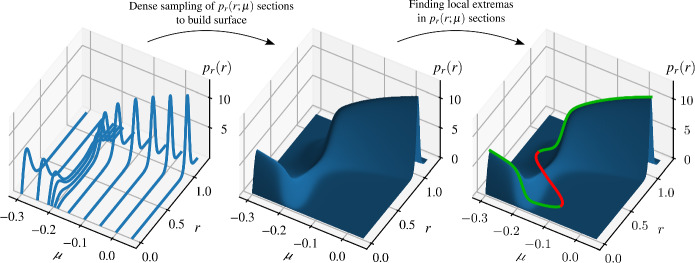
Building the surface $p_r(r;\mu )$ for noise intensity $\sigma _v = 0.1$

**Fig. 5:**
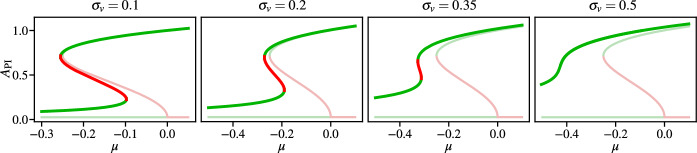
Local extrema $A_{\textrm{PI}}$ in $r$ of $p_r(r;\mu )$ for different noise levels, compared to the bifurcation diagram of the deterministic system (3) (faint lines). The green sections correspond to the characteristic amplitude of the stable, while the red sections correspond to the unstable limit cycles

### Bifurcation diagrams from the PDF

To obtain bifurcation diagrams with respect to the parameter $\mu$ for the stochastic open-loop system (1), the PDFs obtained through the PI method have to be calculated across the parameter range of interest. As depicted in Fig. 4, with dense sampling of $p_r(r,\mu )$ we can build a surface representing the PDF over the $(r,\mu )$ plane. Then, we identify the local minima and maxima of the PDF for different parameter values of $\mu$. These local extrema form S-shaped curves in the $(r,\mu )$ plane. Figure 5 shows these bifurcation curves of the stochastic open-loop system for different $\sigma _v$ noise intensities. The middle section of the S-curves belongs to unstable, the rest to stable near periodic solutions. This means that the bifurcation diagrams are topologically different from the deterministic case. Instead of the stable equilibria, we find stable near periodic solutions in the stochastic system while the unstable equilibria disappear. This means that at the bifurcation point there is no branching as is the case for Hopf bifurcations in deterministic systems. As such, rather than a Hopf bifurcation and a saddle-node bifurcation of the periodic solutions, we observe two saddle-node type bifurcations in the branch of near-periodic solutions. The discrepancy between the characteristic amplitudes of the near-periodic solutions of the stochastic system and the limit cycles in the deterministic system is more pronounced at smaller amplitudes. Also, the discrepancy increases with the noise intensity leading to a reduction in the size of bistable parameter domain. If one increases the noise intensity further, the bistable domain disappears entirely as the noise suppresses the intrinsically nonlinear dynamics of the deterministic core (3). Therefore, we can refer to the level of noise in qualitative terms, i.e. small, moderate and high-intensity noise. By small noise we mean a noise that barely changes the observed dynamic structure of the system, it merely provides a perturbation to the steady state solutions. By moderate intensity noise, we mean a noise that forces the system to switch between the steady states in finite time, independently of the bifurcation parameter. Finally, by high-intensity noise, we mean a noise that completely destroys the bistable behaviour. Based on these definitions, in Fig. 5 we might refer to the noise levels $\sigma _v = 0.1$ to $0.35$ as moderate, and $\sigma _v = 0.5$ as high noise intensity.

**Fig. 6:**
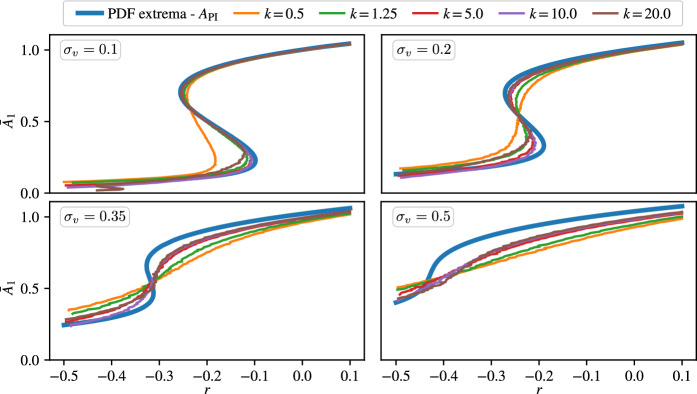
Bifurcation diagrams detected via CBC with different control gains $k$ at different noise intensities $\sigma _v$ compared to the bifurcation curves defined by the extrema of the PDF

### Bifurcation diagrams with CBC

In addition to the characterisation of the steady states of the nonlinear SDEs, we explore what information CBC is capable of extracting from the system. To this end, in Fig. 6 we compare the characteristic amplitudes derived from the PDFs calculated using the PI method with that of obtained by CBC for different control gains.

**Fig. 7:**
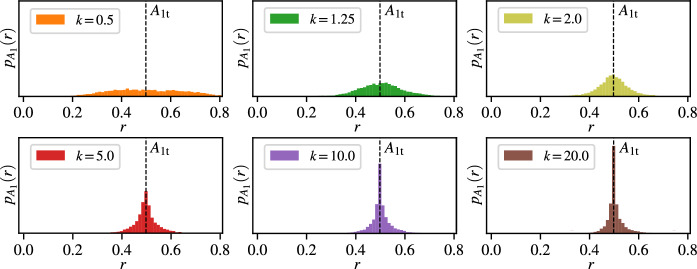
Distributions of the observed characteristic amplitudes $r$ during a single measurement with CBC at $\mu = 0.2$ ($A_{1\textrm{t}} = 0.52$) with a noise intensity $\sigma _v = 0.1$

Even at the smallest noise level $\sigma _v = 0.1$, the control gain $k$ has a strong effect on the obtained bifurcation diagrams in $\bar{A}_1$. The observed characteristic amplitude $\bar{A}_1$ is highly dependent on the control gain $k$. One of the reasons for the discrepancies between the two approaches stems from the fact that CBC uses only the mean of observed characteristic amplitudes. For smaller values of $k$, the control fails to focus the motion around the unstable limit cycle, leading to a highly uncertain and biased identification of $\bar{A}_1$. Figure 7 shows example distributions for $\sigma _v = 0.1$ of the observed characteristic amplitude $\bar{A}_1$ at $\mu = -0.2$ with the target amplitude $(A_{1 \textrm{t}} = 0.52)$ corresponding to the unstable limit cycle, determined from bifurcation diagram from the PDF. Even though the expectation is that with sufficiently strong control, the distribution of the steady state vibration amplitude has a clear peak, this is not the case if the control gain is small, e.g. see the panels with $k=0.5$ or $k=1.0$ where the histograms are wide, and the peak is less clear. Unfortunately, however, it is also not helpful to use an overly strong control, even though this would solve the issue of not having a clear characteristic amplitude in steady state. The reason behind this is that if the control is too strong, then it will stabilise the system around virtually any desired amplitude with little error, leading to an ill-conditioned root-finding problem when we search for the characteristic amplitude where the control is non-invasive. Furthermore, even though the control is designed to be non-invasive at the characteristic vibration amplitude of the system, there is still a nonzero control force in steady state due to the random perturbation to the system which interferes with the nonlinear system dynamics. This interference will eventually result in an error in the steady state amplitude. This is the reason why in Fig. 6 the curves obtained with the largest control gain ($k=20.0$) has a larger error compared to the true amplitude curve than the curves obtained by $k=5.0$ or $k=10.0$. This result also suggests that, for stochastic problems, adaptive control algorithms [39, 40] that address the issue of choosing stabilising control laws in a deterministic setting may need adjustment to address the sensitivity of the identified limit cycles to control strength.

Nevertheless, at the smallest noise level $\sigma _v = 0.1$ in Fig. 6, CBC is capable of extracting these fundamental features of stochastic nonlinear systems provided the control gain is adequate. However, the deterministic framework of CBC has limitations. As one can observe, the agreement between the two types of characteristic amplitudes deteriorates with increasing noise levels. The main gap the CBC method cannot fill is that while it identifies a single characteristic amplitude close to the near periodic solutions, it fails to capture the true PDF of the open-loop system. This is an inevitable consequence of the CBC approach, for which we desire to change the dynamical structure to have an equivalent attractor in place of the unstable near-periodic solution of the uncontrolled system. Therefore, in practice, the amplitudes provided by CBC may be accepted as a ’best guess’ proxy for the vibration amplitudes characteristic to the near periodic solutions in a wide range of experiments where the noise intensity is moderate.

## Conclusions

In this work, we addressed the challenges that arise during the experimental identification of bifurcation diagrams and the continuation of steady state solutions of noisy nonlinear systems with the CBC approach. To this end, we studied the Hopf normal form with 5th-order terms subjected to additive noise for parameters resulting in a subcritical bifurcation and bistability.

The additive noise changes the steady states of the deterministic system such that instead of the limit cycles and the stable equilibria we find near-periodic oscillations. In the noisy case, we defined the unstable limit cycle as the least probable state and the stable limit cycles as the most probable states, corresponding to the local minimum and maxima of the PDF of the oscillation amplitude. With this analysis we confirm that the structure of the bifurcation diagram is significantly altered by the introduction of noise perturbations, even causing the bistable behaviour to disappear at large noise levels.

Next, we search for the limit cycles by adapting the CBC method for continuation of the characteristic amplitudes of the steady states in the stochastic setting. Comparing the bifurcation diagrams identified by CBC to the limit cycles obtained by the PDF-based method highlights the discrepancies between the open-loop and the controlled system. These differences originate from the violation of the non-invasiveness of the control, as random perturbations result in a non-zero control force at the near-periodic solutions. The presence of this noise-intensity-dependent bias of the CBC method in the case of stochastic systems marks the limits of information that can be extracted with this approach.

In the case of the experimental identification of bifurcation diagrams in nonlinear engineering systems, the system is usually subject only to small intensity noise. In the case when this noise is inherent to the system, the CBC method vastly outperforms alternative methods, such as running the system open-loop, and waiting for the discovery of all stable states, both in terms of accuracy and required time. The bottleneck with the open-loop approach in an experimental setting is that steady states that are weakly stable, i.e., are close to the Fold bifurcation, are difficult to detect since even a small perturbation will deter the solution from them, while unstable solutions are practically undetectable. In contrast, the CBC method discovers the bifurcation diagram with high accuracy for these systems, given the control parameter is adequately tuned. Due to the lack of alternative methods to find unstable orbits in experimental settings, one might choose to apply the CBC method in a scenario where the noise driving the system has high intensity. Whether a specific level of noise will be classed as low, moderate or high will be application-dependent. A useful guidance is whether the system exhibits switching between meta-stable states, which clearly indicates significant noise. However, in these cases, the practitioner should be aware when applying the method that CBC is only useful in settings where the system exhibits near-periodic bistable behaviour, and additionally, there might be significant discrepancies between the identified and the actual dynamical structure of the investigated system.

## Figures and Tables

**Algorithm 1:**
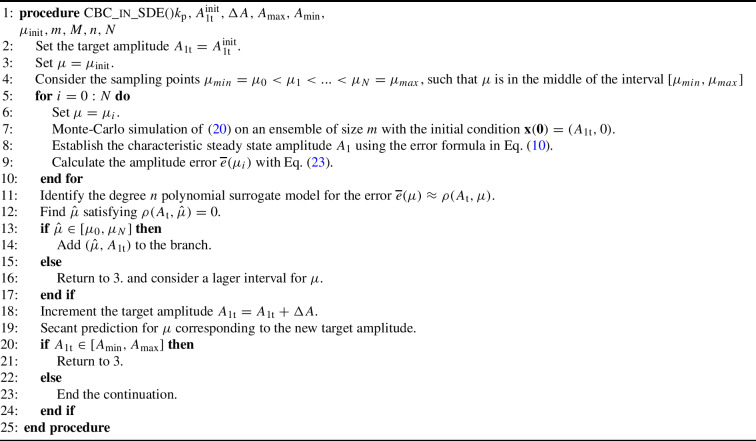
Control-based continuation of the near-periodic solutions of the SDE.

## Data Availability

No relevant data is available for this study.

## Contol-based continuation (CBC)

### Deterministic case

Considering the control target and the solutions in the form of a truncated Fourier series poses an additional challenge in generic autonomous systems, namely, we have to infer the period and the instantaneous phase angle of the self-excited vibrations through measurements. Even in the specific case of the Hopf normal form, where the natural angular frequency is determined by the parameter $\omega$, we still need to identify the phase angle since the steady state periodic orbits are time-invariant. This is resolved by using the instantaneous phase which is calculated as

15 $$\begin{aligned} \phi (t) = \arctan (u(t), v(t)) \end{aligned}$$

for the Hopf normal form. Next, we use this phase angle to obtain the Fourier coefficients of the control target and the steady state solutions. For the Hopf normal form specifically, we also know that the limit cycles are circular periodic orbits in the (*u*, *v*) phase plane. Thus, by choosing *u*(*t*) as the controlled state variable we can consider the steady state periodic solution of the controlled system, the control target and the limit cycles of the open loop system as

16 $$\begin{aligned}  &   u^{*}(t) = A^{*} \cos \phi (t), \end{aligned}$$

17 $$\begin{aligned}  &   u_{1 \mathrm t} (t) = A_\textrm{1t} \cos \phi (t), \end{aligned}$$

18 $$\begin{aligned}  &   u_{1} (t) = A_{1} \cos \phi (t), \end{aligned}$$

namely, we use a single fundamental harmonic term while setting the higher harmonics to zero. In the formulae above, $A^{*}$ refers to the steady state vibration amplitudes in the controlled system, $A_\textrm{1t}$ as the target amplitude, and $A_1$ as the amplitude of the limit cycle of the open-loop system.

With these considerations, we apply an iteration to find the target amplitude $A_{1\mathrm t}$ corresponding to the zero of the amplitude error

19 $$\begin{aligned} e = A_\textrm{1t} - A_1. \end{aligned}$$

We terminate the iteration if $e$ reaches a given tolerance $\epsilon$, i.e. $e < \epsilon$, and we accept the corresponding steady state solution as the limit cycle of the open loop system.

It is important to note that, while CBC can be used in systems with noise-load [26, 35, 41], by assuming the existence of limit cycles in the observed system, the technique is used within an inherently deterministic framework. As such, the interpretation of the captured solutions is only straightforward if the noise has negligible effect on the experiment.

In contrast, a significant noise-load on the system raises several questions. First of all, there is no previous study on how the near-periodic solutions in the SDE and the limit cycles in the underlying deterministic model and the ‘limit cycle’ identified with CBC relate to each other. Or in other terms, whether the non-invasiveness of CBC still holds in a stochastic system. Moreover, the role of the unstable limit cycles is also not rigorously established in a noisy system. In the deterministic framework, unstable limit cycles are frequently used as indicators of the separatrix between the domains of attraction of the two stable solutions. In the presence of noise however the solution may jump between the two domains of attractions due to the external perturbation. Thus, the separatrix cannot act as a perfect barrier.

In what follows, we first discuss the method of CBC to trace periodic orbits in deterministic systems. Then, we present its implementation for the SDE in Eq. (1). We add forcing term to this equation to impose a stabilising control to the system. As such, to study CBC, the following SDE is considered

20 $$\begin{aligned} \textrm{d}{\textbf {x}} = f({\textbf {x}};\mu ) \textrm{d} t + {\textbf {g}}({\textbf {x}}) \textrm{d}{W} + {\textbf {F}}(t)\textrm{d}t, \end{aligned}$$

where the control force is applied in the *u*-direction

21 $$\begin{aligned} \textbf{F}(t) = \begin{pmatrix} F_\textrm{ctrl}(t) \\ 0 \end{pmatrix}. \end{aligned}$$

Considering ${\textbf {g}}(x) \equiv {\textbf {0}}$ in Eq. (20) leads to the deterministic case.

In case of the Hopf normal form, with an appropriately chosen control gain *k*, considering a simple proportional error term as

22 $$\begin{aligned} F_\textrm{ctrl}(t) = k (u_\textrm{t}(t) - u(t)) \end{aligned}$$

provides a stabilising control-law.

### Stochastic case

With the approach described in Sect. 3.1, one can find a near-periodic solution of the system for a given set of parameters. With the continuation algorithm, we would like to trace the related solution-branch as one of the system parameters, referred to as the bifurcation parameter varies. In this example, we survey the system behaviour for different values of the parameter $\mu$. We make use of the fact, that the solution branch in the $(\mu , \overline{A}_1)$ plane has no local extremum in the amplitude. As such, to each amplitude, a unique near-periodic solution corresponds to. Thus, in the continuation algorithm, we fix the target vibration amplitude $A_\textrm{1t}$ while calculating each solution and consider the following zero problem

23 $$\begin{aligned} \overline{e}(\mu ) = A_\textrm{1t} - \overline{A}_1 (A_\textrm{1t}, \mu ). \end{aligned}$$

As mentioned before, this error function is still subject to random perturbations as the vibration amplitude $\overline{A}_1$ is established from a finite sample of the solution PDF, using Monte Carlo simulations. Therefore, derivative-based nonlinear root finding techniques, are not practical in this case. Instead, we take $N+1$ samples between $\mu _{min} = \mu _0< \mu _1<... < \mu _N = \mu _{max}$, such that the interval $[\mu _{min},\mu _{max}]$ includes the suspected solution. Then, we evaluate the error function at each values of $\overline{e}(\mu _j)$ for $j = 0,1,...,N$ and fit an order *n* polynomial surrogate model to the data. Then root-finding is performed on the surrogate model. After identifying a limit cycle, the continuation progresses by incrementing/decrementing the target vibration amplitude $A_\textrm{1t}$. In this study, we used $N=6$ and $n=3$ for the continuation.
